# Pneumococcal nasopharyngeal carriage in children and adults self-confined at home during a COVID-19 national lockdown

**DOI:** 10.1371/journal.pone.0315081

**Published:** 2024-12-05

**Authors:** Pedro Brotons, María Cisneros, Amaresh Pérez-Argüello, Desiree Henares, Aleix Lluansí, Mariona Fernández de Sevilla, Pilar Ciruela, Miguel Blanco-Fuertes, Cristian Launes, Iolanda Jordan, Quique Bassat, Juan José García-García, Carmen Muñoz-Almagro

**Affiliations:** 1 Institut de Recerca Sant Joan de Déu, Hospital Sant Joan de Déu, Esplugues, Barcelona, Spain; 2 CIBER de Epidemiología y Salud Pública (CIBERESP), Instituto de Salud Carlos III, Madrid, Spain; 3 School of Medicine and Health Sciences, Universitat Internacional de Catalunya, Barcelona, Spain; 4 Pediatrics Department, Hospital Sant Joan de Déu, Universitat de Barcelona, Esplugues, Barcelona, Spain; 5 School of Medicine, Universitat de Barcelona, Barcelona, Spain; 6 Agència de Salut Pública de Catalunya, Barcelona, Spain; 7 Pediatric Intensive Care Unit, Hospital Sant Joan de Déu, Universitat de Barcelona, Esplugues, Barcelona, Spain; 8 ISGlobal, Hospital Clínic-Universitat de Barcelona, Barcelona, Spain; 9 Centro de Investigação em Saúde de Manhiça (CISM), Manhiça, Mozambique; 10 InstitucióCatalana de Recerca i Estudis Avançats (ICREA), Barcelona, Spain; University of Cape Town, SOUTH AFRICA

## Abstract

**Background:**

Despite growing evidence of reduced invasive and non-invasive pneumococcal disease attributed to public health measures against the COVID-19 pandemic, the effect of these measures on pneumococcal carriage remains unclear. This study aimed to assess pneumococcal nasopharyngeal carriage among children and adults self-confined at home during the COVID-19 national lockdown in Spain while identifying predictors of pneumococcal carriage in children.

**Methods:**

Household study conducted across the metropolitan area of Barcelona (Spain) between April-June 2020. Nasopharyngeal samples were collected from young children and adults for real-time PCR pneumococcal *lytA* and *wgz* gene detection, quantification, and serotyping, as well as for detection of respiratory viruses.

**Results:**

Among 332 children (median age: 3.1 years [IQR: 1.9–4.0 years]; 59% male) and 278 adults (median age: 38.9 years [IQR: 36.1–41.3 years]; 64% female), pneumococcal carriage rates were 28.3% and 2.5%, respectively. Highly invasive serotypes 3, 7F/7A, and 19A were detected in 14.0% of samples from children carriers. Pneumococcal co-infections with *respiratory syncytial virus* (RSV), *human metapneumovirus* (hMPV), and *influenza virus* (IV) were not identified in children. Attendance to kindergarten before the lockdown (aOR: 2.65; IQR: 1.57–4.47; *p*<0.001) and household crowding (aOR: 1.85; IQR: 1.09–3.15; *p* = 0.02) were independent risk factors for children’s pneumococcal carriage.

**Conclusions:**

Pneumococcal carriage rate among quarantined children during a full COVID-19 lockdown was moderate and correlated with limited presence of highly invasive serotypes and absence of pneumococcal co-infections with RSV, hMPV, and IV. Pre-lockdown daycare and household crowding predisposed children to carriage.

## Introduction

The coronavirus infectious disease 2019 (COVID-19) pandemic had a devasting impact worldwide. In Spain, there were nearly 14 million notified cases and 121,622 confirmed COVID-19-related deaths registered up to June 2023 [1]. A national lockdown was instituted from 14^th^ March until 21^st^ June 2020, imposing strict confinement measures for the population and the closure of all educational, cultural, and leisure facilities [2].

Several nation- and region-wide studies conducted during the COVID-19 pandemic documented a decrease in the incidence of pneumococcal diseases and associated it to the stringent containment measures that were implemented [3–6]. Furthermore, the incidence of respiratory disease caused by viruses commonly involved in pneumococcal disease, such as respiratory syncytial virus (RSV), human metapneumovirus (hMPV), influenza virus (IV), and parainfluenza (PIV) viruses was markedly reduced during the pandemic [4, 7, 8]. Despite growing evidence linking the reduction of IPD and non-invasive pneumococcal disease to the public health measures that were enforced to mitigate the COVID-19 pandemic, the effect of these measures on pneumococcal carriage remains unclear.

The main aim of this study was to determine the rate of pneumococcal nasopharyngeal (NP) carriage and the distribution of serotypes and respiratory viruses among children and adults self-confined in their households during the COVID-19 national lockdown in Spain. Additionally, we aimed to identify risk factors for pneumococcal NP carriage in quarantined children.

## Materials and methods

### Design and study population

This ancillary study was nested within a COVID-19 seroprevalence study that involved 381 volunteer families quarantined in their family households in the densely populated metropolitan area of Barcelona (Catalonia region, Spain) from April 28^th^ to June 3^rd^, 2020 [9]. Families were included in the main study if one parent had been confirmed to be the primary case with COVID-19 in the household at least 15 days before sampling all family members. In the present study, we retrieved nasopharyngeal (NP) samples from every parent that had been identified as the first COVID-19 case in the family and every child aged less than five years from whom a sufficient sample volume was available for analysis.

The 13-valent pneumococcal conjugate vaccine (PCV) was systematically administered to children in Catalonia during the pandemic years, at two, four, and 11 months of age. Systematic anti-pneumococcal vaccination of adults is not mandatory in the region.

### Study variables and data collection

The main outcomes sought were the pneumococcal NP carriage rate and the distribution of serotypes and respiratory viruses detected in children and adults, with an evaluation of risk factors for pneumococcal NP carriage in children as a secondary outcome. At the individual level, the independent variables considered included age, gender, body mass index (BMI)-for-age (in children) and BMI (in adults), presence of respiratory symptoms (in children), detection of respiratory viruses, children’s feeding mode, PCV receipt and time since last PCV dose receipt (in children), and antibiotic intake in the previous 30 days. Household crowding (defined by a proxy, a household occupancy ratio of less than 18 square meters (sqm) per family member according to the minimum housing habitability conditions established in the regional regulations), living with siblings younger than five years of age, face mask use by adults, exposure to parental smoking within the household, and attendance to kindergarten and use of school or public bus transportation before the lockdown were the independent epidemiological variables considered. Data of independent variables were retrieved from the main study’s database.

### Sample collection and microbiological methods

Procedures for sample collection, detection of severe acute respiratory syndrome coronavirus 2 (SARS-CoV-2) and other respiratory viruses (*rhinovirus/enterovirus*, *adenovirus*, *bocavirus types 1/2/3/4*, *coronavirus types 229E/NL63/OC43*, *respiratory syncytial virus* types A/B, *human metapneumovirus*, *influenza virus* types A (subtypes H1/H1N1-2009/H3) and B, and *parainfluenza virus* types 1/2/3/4), detection and quantification of *Streptococcus pneumoniae DNA*, and pneumococcal serotyping have been previously explained [10–12] and are further detailed in S1 File. In brief, NP swabs were collected from participants in their households a minimum of 6 weeks and a maximum of 11 weeks after the start of confinement. Samples were processed at Centro de Regulación Genómica for detection of SARS-CoV-2 and at Hospital Sant Joan de Déu (HSJD) for detection and other respiratory viruses. SARS-CoV-2 reverse-transcriptase PCR was performed according to the CDC-006-00019 protocol. Other respiratory viruses were identified by Allplex™ Respiratory Panels Assays 1, 2 and 3 (Seegene Inc., Seoul, Korea). Surplus samples were bio banked in HSJD and subsequently retrieved for pneumococcal DNA detection and quantification of *lytA* and *wgz* genes by real-time PCR and molecular capsular typing of *lytA*- and *wgz*-positive samples by fluorescence fragment analysis or multiplex qPCR. Serotypes were classified as vaccine serotypes (included in 13-valent, 15-valent, and 20-valent PCVs) and non-vaccine serotypes (not included in PCVs and non-typeable), and as highly and less invasive serotypes. Serotypes 1, 3, 5, 7F/7A, and 19A were considered as highly invasive based on previous evidence [13].

### Statistical analysis

Dichotomous variables were compared by the Chi-square or the Fisher’s exact test. The Student t test or the Mann Whitney test were used for comparison of continuous variables with normal distributions or skewed data, respectively. Pneumococcal NP load was log transformed before analysis. Bivariate analyses were conducted to identify potential associations of pneumococcal NP carriage with independent variables. Independent variables that showed a relationship with carriage at a p-value ≤ 0.10 were considered in multivariate logistic regression analysis. Associations between pneumococcal carriage and risk factors in the multivariate model were measured by adjusted odds ratios (aORs). Significance was set at a p-value of <0.05 and 95% confidence intervals (CIs) were established. Statistical analysis was performed using Stata v.15 software (Stata Corp, TX, US).

### Ethics statement

Adults recruited for the seroprevalence study provided their written informed consent before enrollment. Parents and guardians of participating children provided their written informed consent on their behalf. The HSJD Ethics Committee authorized the main seroprevalence study (Code PIC-59-20), including the use of collected data and samples for further studies nested within it.

## Results

### Characteristics of study population

A total of 620 participants were initially selected for participation. Four participants with invalid results of pneumococcal real-time PCR (no detection of human DNA internal control) were excluded. Six participants whose samples had a valid PCR result but became unpaired after an invalid result for a sample from another pre-selected family member were also excluded. The final study population comprised 610 individuals; 332 children (median age: 3.1 years [interquartile range IQR: 1.9–4.0 years]; 59% male) and 278 adults (median age: 38.9 years [IQR: 36.1–41.3 years]; 64% female) from 278 family households. There were 226 (81.3%) households with one participant child, 50 (18.0%) households with two children, and two households (80.7%) with three children. Among children, 245 out of 332 (73.8%) were between two and four years old. Mean time elapsed since start of confinement until children’s sampling was 64 days (SD: 8.0; range: 47–76 days). No child presented respiratory symptoms. A large proportion (80.1%; 266/332) of children were breastfed for 6 or more months (median breastfeeding time: 6 months; IQR: 4–6 months). Almost all children (99.0%) had received PCVs according to the regional Essential Vaccination Program schedule (median time elapsed since the last vaccine dose receipt: 25.2 months; IQR: 11.0–35.4 months). A low parent-reported exposure (4.3%) to antibiotics in the previous 30 days was observed among children. The median time elapsed since the last antibiotic intake was 78 days (IQR: 42–95 days). Detailed characteristics of the study population are shown in Table 1.

**Table 1:** Individual and epidemiological characteristics of study population.

Variable	Children (n = 332)	Adults (n = 278)
Age, median years (IQR)	3.1 (1.9–4.0)	38.9 (36.1–41.3)
Age group		
≥ 2 years	245/332 (73.8)	-
< 2 years	87/332 (26.2)	-
Gender, male		
Male	196/332 (59.0)	100/278 (36.0)
Female	136/332 (41.0)	178/278 (64.0)
Breastfeeding		
≥ 6 months	266/332 (80.1)	-
< 6 months or formula milk feeding	66/332 (19.9)	-
BMI-for-age		
≥ percentile 3	273/290 (94.1)	-
< percentile 3	17/290 (5.9)	-
BMI (kg/m^2^)		
≥ 18.5 kg/m2	-	265/274 (96.7)
< 18.5 kg/m2	-	9/274 (3.3)
Compliance with pneumococcal vaccination schedule		
Yes	306/309 (99.0)	-
No	3/339 (1.0)	-
Pneumococcal vaccine dose receipt within the previous 30 days		
Yes	6/309 (1.9)	-
No	303/309 (98.1)	-
Antibiotic intake within 30 days before sampling		
Yes	7/164 (4.3)	14/276 (5.1)
No	157/164 (95.7)	262/276 (94.9)
Attendance to kindergarten or school before lockdown		
Yes	154/331 (46.5)	-
No	177/331 (53.5)	-
Use of school bus before lockdown		
Yes	32/323 (9.9)	-
No	291/323 (90.1)	-
Household occupation rate		
≥ 18 sqm	212/325 (65.2)	184/272 (67.7)
< 18 sqm	113/325 (34.8)	88/272 (32.3)
Siblings aged < 5 years		
Yes	108/332 (32.5)	-
No	224/332 (67.5)	-
Face mask use by COVID-19 primary case in the household		
Always	-	88/278 (31.6)
Sometimes or never	-	190/278 (68.4)
Exposure to parental smoke in the household		
Yes	63/332 (19.0)	-
No	269/332 (81.0)	-
Parent smoking habit		
Yes	-	50/278 (18.0)
No	-	228/278 (82.0)

Variables expressed as proportions (%), unless otherwise stated

Abbreviations: IQR, interquartile range; BMI, body mass index; NP, nasopharyngeal; sqm, square meters; COVID-19, coronavirus disease 2019

### Pneumococcal nasopharyngeal carriage and serotype distribution

**Fig 1:**
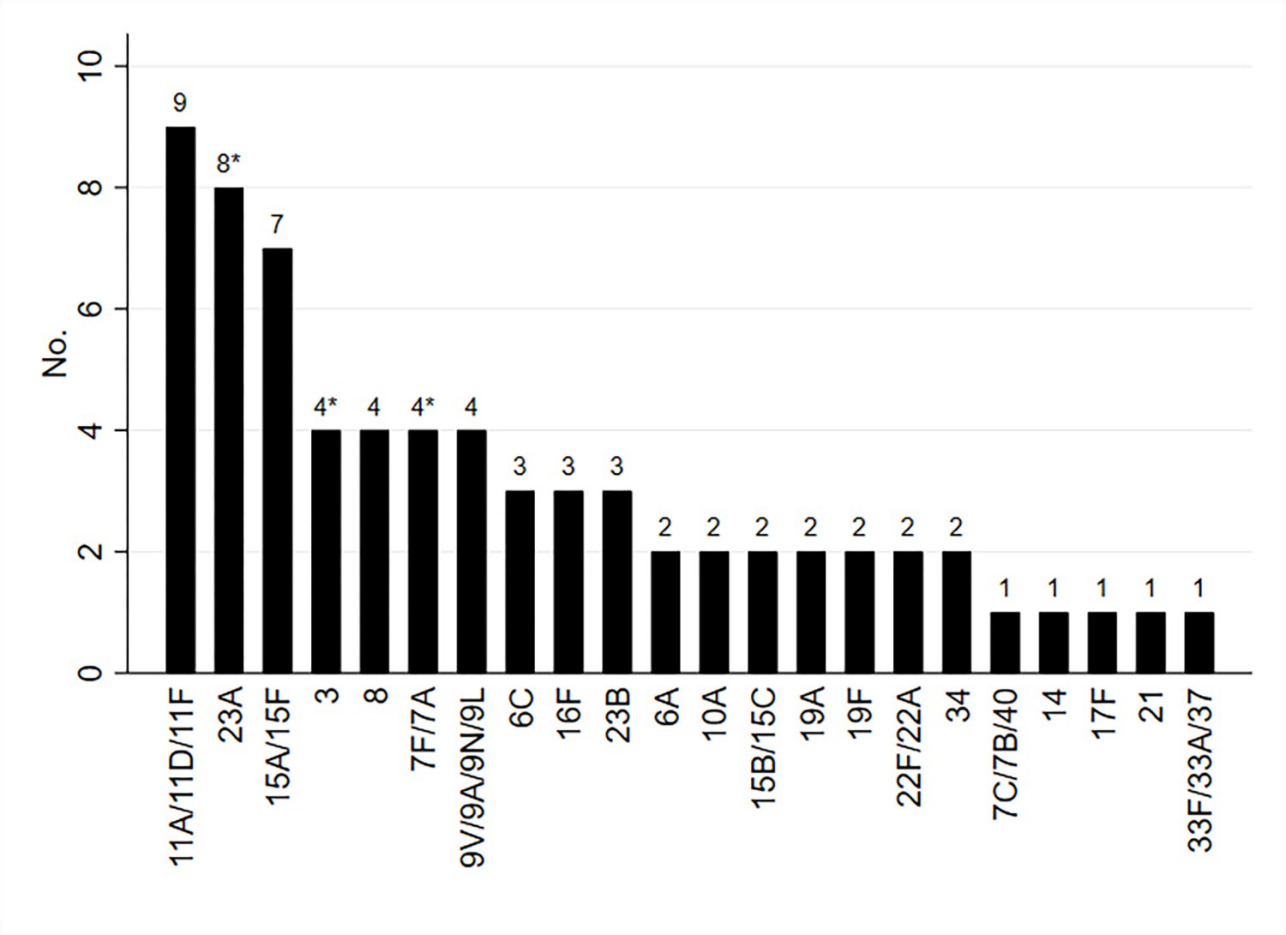
Distribution of pneumococcal serotypes in children and adults. * Including one serotype 3, one serotype 7F/7A, and one serotype 23A detected in adults.

Ninety-four out of 332 (28.3%) children were found to be pneumococcal NP carriers, with a median pneumococcal DNA density of 3.99 log_10_ copies/ml (IQR: 3.38–4.58 log_10_ copies/ml). Out of 52 households with two or more children, 17 (32.7%) included both children carriers and non-carriers while in 11 (21.2%) all children were carriers. No child that received antibiotics in the previous 30 days was observed to be a carrier. Serotyping was possible in 59*lytA*-positive samples, including 57 from children and two from adults. Serotypes 11A/11D/11F (n = 9), 15A/15F (n = 7), and 23A (n = 7) were the most prevalent serotypes carried by children. Highly invasive serotypes 3 (n = 3), 7F/7A (n = 3), and 19A (n = 2) were identified in eight (14.0%) samples from children carriers. Co-detection by two serotypes was found in eight (14.0%) children samples containing serotypes 7F/7A and 23 A (two samples), 3 and 23A, 6A and 6C, 7F/7A and 15A/15F, 9VANL and 23 A, and 15A/15F and 33FA/37, respectively. A total of 70/94 (74.4%) of children carried non-vaccine serotypes, including five carriers of both non-vaccine and vaccine serotypes. Serotypes were identifiable in four couples of sibling carriers in four out of the 11 households where all children were carriers. Only in one household both siblings carried the same serotype, which was the less invasive serotype 34. Seven out of 278 (2.5%) adults were identified as carriers, with a median NP carriage load of 3.01 log_10_ copies/ml (IQR: 2.19–4.26 log_10_ copies/ml). Among the two samples from adult carriers that could be serotyped, highly invasive serotype 3 was detected alone in one while highly invasive serotype 7F/7A was co-detected with less invasive serotype 23A in the other. See Table 2 for pneumococcal NP carriage, colonization density, and serotype classifications in children and adults, and Fig 1 for serotype distribution.

**Table 2:** Pneumococcal nasopharyngeal carriage rates and serotype and respiratory viral detection in children and adults.

Variable	Children (n = 332)	Adults (n = 278)
NP *Sp* carriage	94/332 (28.3)	7/278 (2.5)
≥ 2 years	70/245 (28.6)	-
< 2 years	24/87 (27.6)	-
NP *Sp* DNA load, median log _10_ copies/ml (IQR)	3.99 (3.38–4.58)	3.01 (2.19–4.26)
≥ 2 years	3.85 (3.09–4.56)	-
< 2 years	4.25 (3.69–4.65)	-
NP *Sp* serotype detection	57/94 (60.6)	2/7 (28.6)
Single serotype	49/57 (86.0)	1/2 (50.0)
2 serotypes	8/57 (14.0)	1/2 (50.0)
Vaccine coverage of NP *Sp* serotypes		
Not covered	65/94 (69.1)	5/7 (71.4)
Covered	24/94 (25.5)	1/7 (14.3)
Co-detection of vaccine and non-vaccine serotypes	5/94 (5.3)	1/7 (14.3)
Invasiveness potential of NP *Sp* serotypes		
Less invasive	49/57 (86.0)	-
Highly invasive	4/57 (7.0)	1/2 (50.0)
Co-detection of highly and less invasive serotypes	4/57 (7.0)	1/2 (50.0)
Respiratory virus detection in samples	119/332 (35.8)	103/278 (37.1)
*Adenovirus*	12/119 (10.1)	-
*Bocavirus*	35/119 (29.4)	4/103 (3.9)
*Coronavirus*	1/119 (0.8)	-
*Influenza virus B*	1/119 (0.8)	-
*Parainfluenza virus 1*	6/119 (5.0)	1/103 (1.0)
*Parainfluenza virus 2*	3/119 (2.5)	-
*Parainfluenza virus 3*	1/119 (0.8)	-
*Parainfluenza virus 4*	1/119 (0.8)	-
*Respiratory syncytial virus B*	1/119 (0.8)	-
*Rhinovirus/enterovirus*	72/119 (60.5)	15/103 (14.6)
SARS-CoV-2	34/119 (28.6)	99/103 (96.1)*
No. of respiratory viruses detected	119/213 (35.8)	103/278 (37.1)
Two or more	38/119 (31.9)	14/103 (13.6)
One	81/119 (68.1)	89/103 (86.4)

Variables expressed as proportions (%), unless otherwise stated

* SARS-CoV-2 detection in adult primary cases ≥ 15 days after first detection

Abbreviations: IQR, interquartile range; NP, nasopharyngeal; *Sp*, *Streptococcus pneumoniae*; SARS-CoV-2, severe acute respiratory syndrome coronavirus 2

### Detection of respiratory viruses

A total of 119 out of 332 (35.8%) samples from children and 103 out of 278 (37.1%) samples from adults tested positive for respiratory viruses. Co-infections with two or more viruses were found in 38/119 (31.9%) of samples from children and in 14/103 (13.6%) of samples from adults. Among children, the predominant viral species detected was RV/EV (60.5%), followed by BoV (29.4%) and SARS-CoV-2 (28.6%). Out of the 94 samples collected from children carriers, 40 (42.6%) were co-infected with respiratory viruses, including 30 (31.9%) with RV/EV, 12 (12.8%) with BoV, and 9 (9.6%) with SARS-CoV-2. Co-infection of pneumococcus with viruses commonly associated with pneumococcal disease was infrequent, in only four (4.3%) out of 94 samples containing PIV-2 (n = 3) and PIV-1 (n = 1). No co-infections with RSV, hMPV, or IV were identified among children carriers. As expected, SARS-CoV-2 (96.1%) was highly predominant in samples from adults confirmed as COVID-19 primary cases, with other viral species having a minor presence in this age group. Only one co-infection of pneumococcus with a viral species, specifically SARS-CoV-2, was detected among adult carriers. See Table 2 for details of distribution of respiratory viruses in children and adults.

### Association between pneumococcal nasopharyngeal carriage and independent variables

A significant difference in pneumococcal NP carriage rate was identified between children and adults (28.3% *vs*. 2.5%; *p* < 0.001). However, there were no significant differences in median pneumococcal DNA load between the two groups (3.99 *vs*. 3.01 log_10_ copies/ml, *p* = 0.09). Pneumococcal NP carriage rates were similar in children aged two or more years (70/245, 28.6%) and younger children (24/87, 27.6%, *p* = 0.86). The difference in median pneumococcal DNA load between the two groups of children was not significant, either (3,85 *vs*. 4,25 log_10_ copies/ml, *p* = 0.27). Among children, pneumococcal NP carriage was found to be independently associated with attendance to kindergarten before the national lockdown (OR, 2.86; *p* < 0.001), household crowding represented by a household occupancy rate below 18 sqm/person (OR, 1.99; *p* < 0.01), and detection of two or more respiratory viruses *versus* none (OR, 2.14; *p* < 0.04). In addition, a tendency to statistical significance was found for children living with siblings aged less than five years (OR: 1.63; *p* = 0.06). When all these variables associated with pneumococcal NP carriage under a threshold *p*-value of 0.10 were introduced into a multivariate regression model, attendance to kindergarten prior to the lockdown (adjusted odds ratio aOR: 2.65; IQR: 1.57–4.47; *p* < 0.001) and a household occupancy rate below 18 sqm/person (aOR: 1.85; IQR: 1.09–3.15; *p* = 0.02) remained as significant and independent risk factors for pneumococcal carriage in children. See Table 3 of factors for pneumococcal nasopharyngeal carriage in children.

**Table 3:** Factors for pneumococcal nasopharyngeal carriage in children.

	Univariate analysis			Multivariate analysis		
Variables	OR	95% CI	*p* value	aOR	95% CI	*p* value
Age group, ≥ 2 *vs*. < 2 years	1.05	0.61–1.81	0.86			
Gender, female *vs*. male	1.24	0.76–2.00	0.39			
Breastfedding, ≥ 6 *vs*. < 6 months or formula milk feeding	1,03	0.98–1.10	0.25			
BMI-for-age, < 3 *vs*. ≥ 3 percentile	0.86	0.27–2.72	0.80			
Pneumococcal vaccine dose received within the previous 30 days, yes *vs*. no	1.22	0.22–6.79	0.82			
*Adenovirus* detection, yes *vs*. no	1.85	0.57–5.99	0.30			
*Bocavirus* detection, yes *vs*. no	1.37	0.65–2.88	0.41			
*Rhinovirus/enterovirus* detection, yes *vs*. no	2.19	1.27–3.78	<0.01	2.27	0.89–5.79	0.09
SARS-CoV-2 detection, yes *vs*. no	0.90	0.40–2.01	0.80			
No. of respiratory viruses detected						
Two or more *vs*. none	2.14	1.05–4.37	0.04	0.93	0.30–2.90	0.91
One *vs*. none	1.24	0.70–2.19	0.46	0.78	0.35–1.74	0.55
**Attendance to kindergarten or school before lockdown, yes *vs*. no**	**2.86**	**1.74–4.70**	**<0.001**	**2.65**	**1.57–4.47**	**<0.001**
Use of school or public bus transportation before lockdown	1.34	0.62–2.89	0.46			
**Household occupation rate, < 18 *vs*. ≥ 18 sqm**	**1.99**	**1.21–3.26**	**0.01**	**1.85**	**1.09–3.15**	**0.02**
Siblings aged < 5 years, yes *vs*. no	1.63	0.99–2.68	0.06	1.34	0.78–2.31	0.29
Face mask use by COVID-19 primary case in the household, always *vs*. sometimes/never	0.89	0.53–1.49	0.65			
Passive smoke exposure in the household, yes *vs*. no	1.12	0.61–2.04	0.72			
Pneumococcal carriage in a family member, yes *vs*. no	1.45	0.88–2.40	0.15			

Abbreviations: OR, odds ratio; aOR, adjusted odds ratio; CI, confidence interval; BMI, body mass index; NP, nasopharyngeal; sqm, square meters; COVID-19, coronavirus disease 2019; SARS-CoV-2, severe acute respiratory syndrome coronavirus 2

## Discussion

Very few studies have focused on evaluating pneumococcal carriage in children during a full lockdown in response to the COVID-19 pandemic. In this household study, we determined the pneumococcal NP carriage rate in children and adults that were sampled in a continuous and uninterrupted manner during a national lockdown in Spain. Pneumococcal carriage rate (28.3%) was found to be moderate in children, despite increased opportunities for child-child pneumococcal transmission in quarantined family households. Detection of highly invasive serotypes was relatively low (14.0%) among children carriers, as well as detection of respiratory viruses commonly associated with pneumococcal disease (4.3%).

The moderate prevalence rate of pneumococcal NP carriage (28.3%) found in children was similar to the rate of 23.5% reported in a previous study conducted by our group with 502 asymptomatic children in the same geographical area before the pandemic [14]. These results might suggest that pneumococcal carriage in children could have remained stable and largely unaffected by the public health measures adopted against the spread of COVID-19. Such finding is intriguing, since one could expect that self-confinement of families in the enclosed space of their households for over two months would increase opportunities for pneumococcal transmission between children.

Results of other studies also pointed to the persistence of pneumococcal carriage in pediatric populations during the COVID-19 pandemic. In southern Israel, the monthly rates of pneumococcal carriage in young healthy children visiting a hospital or hospitalized without respiratory infections were only slightly reduced in 2020–2021 (the rate series was interrupted in April-May 2020, during the full lockdown in that country) in comparison to the mean carriage rate observed in 2016–2019 [4]. In France, an analysis of ambulatory and hospital-based pediatric data from multiple national surveillance systems revealed no substantial change in pneumococcal carriage rates after the implementation of social restriction measures while the incidence of IPD decreased [5]. In Belgium, where a lockdown was enacted in mid-March 2020, a comparison between monthly pneumococcal carriage rates in young children attending daycare centers from November to March in 2017–2019 and 2020–21, respectively, showed a sustained and high rate of pneumococcal carriage across the two periods [15].

The low pneumococcal carriage rate (2.5%) determined in our study among mid-age adult carriers was congruent with pneumococcal carriage rates reported for other adult groups during the pandemic. In Serbia, a pneumococcal carriage prevalence of 3.1% was reported in adults aged 50 years and older during 2021 [16]. Similarly, elder adults had a carriage rate of 4.2% in Denmark during the initial lockdown, which was three times lower than in the pre-lockdown period [17]. Of note, several studies conducted in high-income countries had estimated low colonization rates among adults in the pre-pandemic years [18, 19]. Overall, these results may indicate that pneumococcal carriage stabilization in developed countries during the pandemic could have occurred not only in children but also in adults.

Detection of PIV was low and RSV, hMPV, and IV were not even detected in children, whereas RV/EV was frequently identified in this age group. Indeed, several studies reported a sharp decline in the circulation of RSV, hMPV, IV, and PIV, all of them viruses commonly associated with pneumococcal disease in the pre-pandemic years, in contrast with the unrestricted circulation of RV and ADV [4, 7, 8]. The correlation between decreased circulation of RSV, IV, and hMPV, and decreased pneumococcal disease observed during the pandemic reveals that these viruses may play an important role in the pathogenesis of disease caused by pneumococcus, as previously suggested [20]. It is also worth noting that the RSV season typically ends in Spain by March, and therefore a low detection of this viral species could be expectable during our period of study.

The composition of the NP microbiota, the ecological niche of pneumococcus, could also influence the pathways to colonization and pneumococcal disease. In previous studies, we identified a shift in the relative abundance of *Streptococcus* and other commensal bacterial genera present in the nasopharynx of healthy children. Before the pandemic, *Moraxella* was found to be the predominant compositional genera (27.6%) in the NP microbiota of healthy children, followed by *Haemophilus* (16.7%) and *Streptococcus*, with minor contribution of *Dolosigranulum* (1.1%) and *Corynebacterium* (0.1%) [21]. Conversely, commensal *Corynebacterium* (25.7%) and *Moraxella* (21.7%) predominated in the nasopharynx of children self-confined during the pandemic, also characterized by a noticeable abundance of *Dolosigranulum* (16.4%) and limited abundance of *Streptococcus* (5.3%) and *Haemophilus* (5.2%) [22]. In this regard, some studies have suggested that *Dolosigranolum* and *Corynebacterium* species could inhibit pathogenic bacteria and promotea certain degree of protective immune responses whereas *Streptococcus* and *Haemophilus* genera, as well as some *Moraxella* species, have been associated with severe acute respiratory infection and asthma [23–25].

Attendance of children to kindergarten before the lockdown and household crowding were identified as risks factors for children’s pneumococcal carriage. Both factors have extensively been reported to predispose for carriage in children, as documented in a recent systematic study [26]. Of note, previous cohort studies estimated a mean or median duration of 31 and 61 days [27, 28], respectively, for children carriage. Interestingly, time elapsed between the lockdown start date and the date of sampling for children carriers ranged from 6 to 11 weeks, overlapping with estimates of carriage duration previously reported. In addition, we observed a relatively low carriage of highly invasive serotypes and only identified a couple of siblings in one household who carried the same serotype. Moreover, having young siblings, a well-known factor favoring pneumococcal carriage, was not determinant for pneumococcal carriage in children. Overall, these findings support the hypothesis that most children became colonized before the lockdown, probably through encounters with other children in the kindergarten rather than through intra-household interactions with their siblings during the lockdown.

The study has several limitations. First, it was conducted in a population characterized by specific sociodemographic and household conditions in a delimited geographic area during the early pandemic period. Any extrapolation of our findings to other geographic contexts or later pandemic periods should be made with caution. However, as discussed previously, other studies conducted in diverse European settings during different pandemic periods reported similar results of moderate pneumococcal carriage in children, which supports the validity of our results. Second, some of the children’s NP samples did not contain enough volume to determine pneumococcal carriage. However, as disregarded samples were randomly distributed across family households, we estimate that any potential selection bias would have a minor impact. Third, almost half of parents were not able to remember if their children had taken antibiotics recently, a factor that could reduce the pneumococcal carriage rate. Given the lack of respiratory symptoms among quarantined children, we speculate that the likelihood of these parents giving antibiotics to their children may be low and similar to the low proportion of antibiotic use reported by parents who were able to report this item. Finally, we determined pneumococcal NP carriage only in first COVID-19 adult cases in households. Since pneumococcal colonization could be associated with SARS-CoV-2 positivity, a potential bias in the adult pneumococcal colonization rate cannot be disregarded.

In conclusion, we observed a moderate pneumococcal NP carriage rate in children self-confined with their families in urban family households during the COVID-19 national lockdown in Spain that correlated with relatively low children’s carriage of highly invasive serotypes and absence of pneumococcal co-infection with RSV, hMPV, and IV. Attendance to kindergarten prior to the lockdown and household crowding were found to be risk factors for pneumococcal carriage in children. Further studies including NP microbiota analysis are needed to gain a deeper understanding of the evolution of pneumococcal carriage in children before and during the pandemic.

## Supporting information

S1 File Sample collection and microbiological methods. (DOCX)
