# Supplementary material for: Pneumococcal nasopharyngeal carriage in children and adults self-confined at home during a COVID-19 national lockdown
Source: PLoS One. 2024 Dec 5;19(12):e0315081. doi: 10.1371/journal.pone.0315081 (PMC11620455; doi:10.1371/journal.pone.0315081)
Supplement: S1 File — (DOCX) [file pone.0315081.s001.docx]

**Supporting information. Sample collection and microbiological methods**

NP swabs were collected from participants in their households a minimum of 6 weeks and a maximum of 11 weeks after the start of confinement. Samples were processed at the Centro de Regulación Genómica for detection of SARS-CoV-2 and at the Support Laboratory for Molecular Surveillance of IPD in Catalonia in Hospital Sant Joan de Déu (HSJD) for detection and other respiratory viruses.

SARS-CoV-2 reverse-transcriptase PCR was performed according to the CDC-006-00019 protocol, which considers detection of the N1 or N2 SARS-CoV-2 genes and the RNase P human gene as internal control. Other viral respiratory infections were identified in NP samples by Allplex™ Respiratory Panels Assays 1, 2 and 3 (Seegene Inc., Seoul, Korea) targeting *rhinovirus/enterovirus* (RV/EV), *adenovirus* (AdV), *bocavirus* (BoV), *coronavirus* (CoV), RSV types A and B, hMPV, IV types A and B, and PIV types 1, 2, 3 and 4.

NP samples that were bio-banked during the main study and had sufficient volume for further analysis were retrieved for pneumococcal detection, quantification, and serotyping in HSJD. Automated DNA extraction was performed using magnetic silica beads. Five micro liters of the DNA extract were added to the PCR reaction mix, followed by performance of a quantitative real-time PCR targeting the *lytA* gene of *S*. *pneumoniae* and the internal control targeting RNaseP of human cells. The sequence of primers and probes recommended by CDC for both the pathogen and the internal control were used (<http://www.cdc.gov/meningitis/lab-manual/chpt10-pcr.html>). DNA was amplified with the QuantStudio™ 5 Real-Time PCR System. Molecular capsular typing of *lytA*-positive nasopharyngeal samples was performed using fluorescence fragment analysis targeting 40 serotypes/serogroups. Alternatively, *S. pneumoniae* serotypes were identified by multiplex qPCR when the PCR cycle threshold (Ct) value was greater than 30. The following serotypes and subtypes were detected: 1, 2, 3, 4, 5, 6A/6B, 6C, 7F/7A, 7C/7B/40, 8, 9V/9A, 9N/9L, 10A, 10F/10C/33C, 11A/11D/11F, 12F/12A/44/46, 13, 14, 15A/15F, 15B/15C, 16F, 17F, 18A/18B/18C/18F, 19A, 19F, 20, 21, 22F/22A, 23A, 23B, 23F, 24A/24B/24F, 31, 33F/33A/37, 34, 35A/35C/42, 35B, 35F/47F, 38/25F, and 39. Serotypes were classified as vaccine serotypes (included in 13-valent, 15-valent, and 20-valent PCVs) and non-vaccine serotypes (not included in PCVs and non-typeable), and as highly and less invasive seotypes. Serotypes 1, 3, 5, 7F/7A, and 19A were considered to be highly invasive based on previous evidence. When two serotypes or serotype subtypes could not be differentiated from each other and any of the serotypes or subtypes was invasive and/or covered by PCVs, we classified the indistinguishable serotype as an invasive and/or vaccine serotype. If more than two serotypes or serotype subtypes could not be differentiated, we classified the serotype as non-invasive and/or non-vaccine serotype.
